# Accelerating organic solar cell material's discovery: high-throughput screening and *big data*†

**DOI:** 10.1039/d1ee00559f

**Published:** 2021-04-23

**Authors:** Xabier Rodríguez-Martínez, Enrique Pascual-San-José, Mariano Campoy-Quiles

**Affiliations:** Institut de Ciència de Materials de Barcelona, ICMAB-CSIC, Campus UAB 08193 Bellaterra Spain m.campoy@csic.es

## Abstract

The discovery of novel high-performing materials such as non-fullerene acceptors and low band gap donor polymers underlines the steady increase of record efficiencies in organic solar cells witnessed during the past years. Nowadays, the resulting catalogue of organic photovoltaic materials is becoming unaffordably vast to be evaluated following classical experimentation methodologies: their requirements in terms of human workforce time and resources are prohibitively high, which slows momentum to the evolution of the organic photovoltaic technology. As a result, high-throughput experimental and computational methodologies are fostered to leverage their inherently high exploratory paces and accelerate novel materials discovery. In this review, we present some of the computational (pre)screening approaches performed prior to experimentation to select the most promising molecular candidates from the available materials libraries or, alternatively, generate molecules beyond human intuition. Then, we outline the main high-throuhgput experimental screening and characterization approaches with application in organic solar cells, namely those based on lateral parametric gradients (measuring-intensive) and on automated device prototyping (fabrication-intensive). In both cases, experimental datasets are generated at unbeatable paces, which notably enhance *big data* readiness. Herein, machine-learning algorithms find a rewarding application niche to retrieve quantitative structure–activity relationships and extract molecular design rationale, which are expected to keep the material's discovery pace up in organic photovoltaics.

## Abstract 2

This review article presents the state-of-the-art in high-throughput computational and experimental screening routines with application in organic solar cells, including materials discovery, device optimization and machine-learning algorithms.

## Graphical abstract

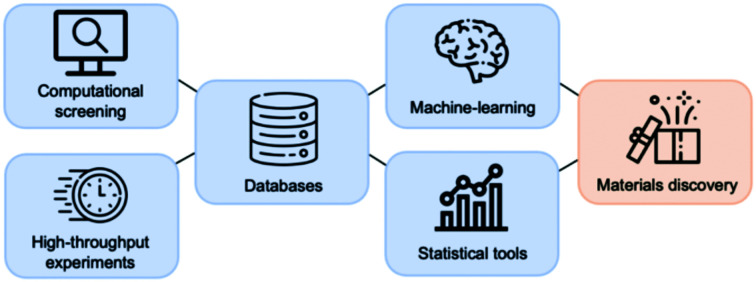

**Broader context**

Organic photovoltaic (OPV) materials discovery of novel small molecular acceptors and low band gap donor polymers has recently propelled power conversion efficiencies to figures approaching 20%. High-throughput screening routines, deployed both *in silico* and experimentally, constitute the foremost strategies behind the unsparing increase in photovoltaic performance and materials discovery pace experienced by this technology in the last few years. In this review, we present the latest advances in high-throughput combinatorial workflows aimed to accelerate OPV materials discovery and device optimization beyond classical one-sample-at-a-time experimentation. In parallel, these approaches have fostered the generation of big datasets at unprecedented paces, thus rendering the OPV scene as an ideal application niche for advanced statistical and artificial intelligence (AI) algorithms. In this work, we also advent distinct synergic usages of AI in OPV to extract hidden patterns from computational and experimental datasets, as well as to orchestrate experimental execution in next-generation robotized and self-driven laboratories. Arguably, these are considered as the game-changing material screening routines defining the OPV technology roadmap in the upcoming decade.

## Introduction

Organic photovoltaics (OPVs) are certainly gaining momentum: the 20% efficiency milestone in single-junction devices is nowadays closer than ever.^1^ Indeed, power conversion efficiencies (PCEs) in excess of 18% have already been demonstrated under 1 sun and even higher under indoor illumination.^2–4^ This is taking dream traits of OPVs closer to reality, including the promise of a flexible and lightweight photovoltaic technology with tunable degrees of transparency and colour^5^ at extremely low energy payback times.^6^

**Fig. 1:**
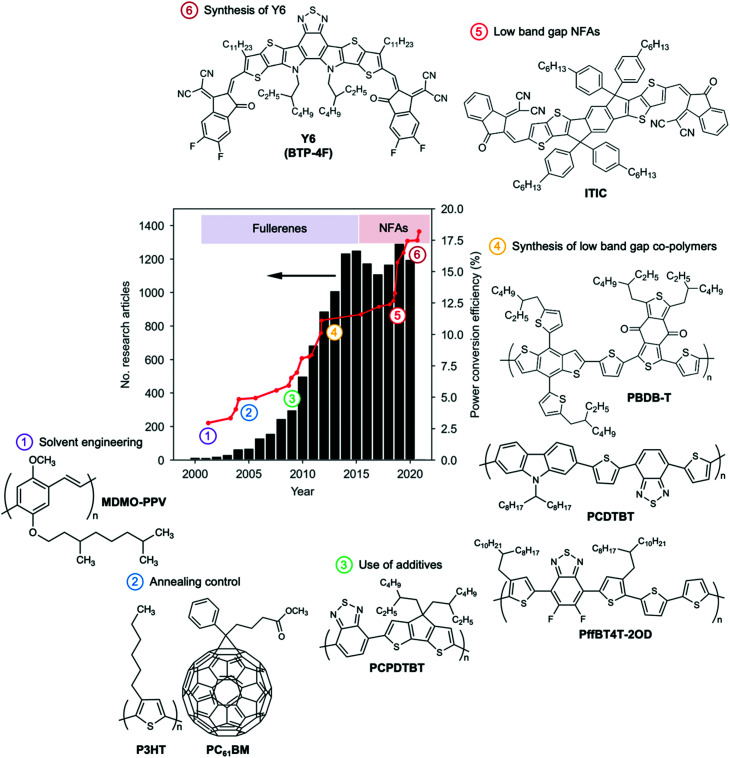
The development stages of OPV illustrated as the number of original research articles (excluding review articles) dealing with ‘organic solar cells’ and ‘organic photovoltaics’ published during the last 20 years and time-evolution of the certified PCE in OPV lab-cells. Important milestones related to processing, active layer morphology and chemical design of materials enabled the OPV technology to keep up growing until reaching PCE figures unthinkable only ten years ago. The search on the number of research articles was performed in the Web Of Science engine using the following keywords: AB = (“organic solar cell*” OR “organic photovoltaic*” NOT (“perovskite” OR “dye-sensitized” OR “DSSC” OR “diode” OR “photodetector” OR “quantum dot*”)) while selecting articles, proceedings and letters amongst the resulting citations (search performed on January 5th, 2021). PCE data was adapted from the NREL Best Research-Cell Efficiency Chart.^11^

Many excellent reviews exist that describe the operational principles and progress of the OPV field over the years.^7–10^ Here we would like to contextualize the field by summarizing the different historical stages in terms of PCEs, as shown in Fig. 1.^11^ The initial stage comprises the first decade of the 21st century in which most of the fundamental physical concepts were established, basic geometries explored and the role of the active layer morphology revealed.^7^ Then, the introduction of low band gap donor polymers to substitute more classical materials such as polythiophenes, took PCEs up to the 10% regime.^12^ More recently, the vertiginous increase in efficiency over the last five years is the result of the irruption of non-fullerene acceptors (NFAs) in the OPV scene.^13,14^ Every year, an increased number of NFAs are being synthesized targeting improved light absorption capabilities (leading to large short-circuit current densities, *J*_sc_) as well as fine-tuned energy levels (resulting in optimized open-circuit voltages, *V*_oc_).

An important feature of NFAs (*cf.* fullerenes) relates to their chemical flexibility, which enables proper adjustment of energy levels, band gaps, solubility and crystallinity, resulting in an endless catalogue of potentially high-performing NFA candidates.^9,13,14^ On the other hand, push–pull donor copolymers are synthetically flexible as well, thus the number of donor:acceptor pairs waking OPV interest up is seemingly infinite. Clearly, the efforts of the community are also increasing from a few hundred papers per year, to over a thousand in recent years, especially after breaking the 10% PCE milestone back in 2012 (Fig. 1). Indeed, there are many open questions still to be addressed together with ever increasing efficiency values, especially regarding improving stability, reducing toxicity, limiting cost, and demonstrating scalability. Thus, how can one effectively identify the system that will take efficiency, stability and cost beyond the state-of-the-art? How can one find the “electrifying” needle in the materials haystack?

**Fig. 2:**
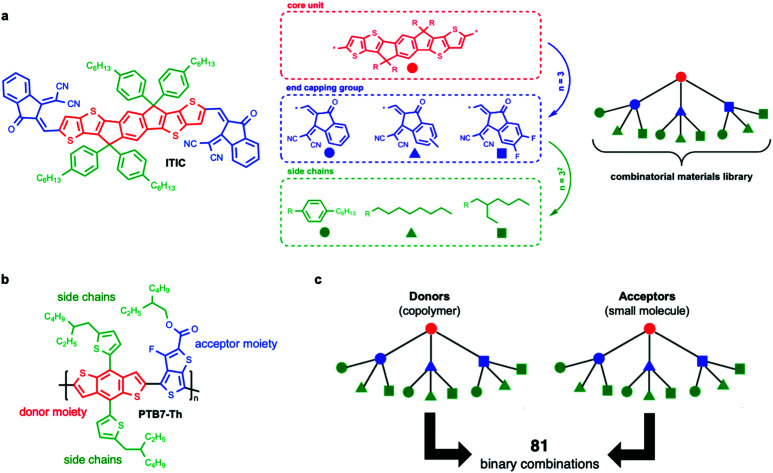
The structural variability of organic semiconductors, and hence the tunability of their optoelectronic properties, is enormous, as exemplified in the following case scenario. (a) The workhorse ITIC acceptor molecule is composed of a seven-ring conjugated fused core as the central unit (indacenodithieno[3,2-*b*]thiophene, IT, colored in red). Then, dissimilar ITIC derivatives can be identified depending on the type of end capping groups attached (choosing amongst three, colored in blue) and grafted side chains (choosing amongst three, colored in green). As depicted on the right, in this depth-limited combinatorial tree example (where each type of end-capping group and side chain has been assigned to different colored symbols), we already identify 3^2^ distinct materials in the corresponding library. Some of the proposed molecules have already been synthesized, characterized and used in organic solar cells with excellent results (namely ITIC, ITIC-M, ITIC-4F, ITIC-C_8_ and ITIC-C_2_C_6_); others have not been reported yet in the literature. (b) A similar combinatorial materials library applies to conjugated copolymers such as PTB7-Th, in which the type of electron-donating moiety (red), electron-withdrawing moiety (blue) and grafted side chains (green) can also be varied systematically. (c) When building an organic solar cell as per the architecture of binary bulk heterojunctions, up to 81 different donor:acceptor combinations are possible. Whether the unexplored systems are superior or not to those already known is a demanding problem that computational screening, high-throughput experimentation and AI algorithms are currently trying to unravel more systematically.

As a first step, computational screening of materials taps on the many years of experience in molecular design to provide guidance for novel compounds. These methods enable the *in silico* exploration of extremely large material libraries, suggesting promising material candidates. While this approach narrows the possibilities, entire families of compounds have to be synthetized and tested experimentally in combination with a large number of potential partners and device geometries (Fig. 2a and b). Therefore, an additional challenge is to experimentally screen large bodies of molecules, processing parameters and geometries.

For any given donor:acceptor pair, researchers have encountered a vast configurational landscape shaping the device performance. Therein, the location of the optimum is determined by a combination of (1) intrinsic material properties such as energy level alignment, miscibility, optical extinction or exciton diffusion length;^15^ (2) extrinsic conditions related to the film processing such as casting temperature or ink formulation; and (3) device features such as the active layer thickness or the donor:acceptor blend ratio forming the OPV blend. Due to its large complexity, investigating efficiently and completely the parametric space becomes unaffordable following traditional one-variable-at-a-time methods (Edisonian approaches, see below), in which each of the involved parameters is varied individually. These facts, together with the ever-growing catalogue of OPV materials and combinations thereof (Fig. 2c), result in a vast combinatorial scenario where classical screening methodologies (in which donor:acceptor pairs are tested individually until nailing the optimum performance) are no longer time and cost effective. As a consequence, the materials library grows faster than the human workforce and resources do. In order to tackle this scenario, efficient screening and optimization workflows have naturally emerged and have been implemented in OPV and thin film technologies alike, thus overcoming the throughput limitations of classical sample-by-sample approaches.

The concept of high-throughput experimentation was first introduced in 1995 to drive the discovery of superconducting materials by exploiting combinatorial stoichiometric libraries.^16^ Since then, the development of combinatorial libraries has been progressively extended to other fields within materials science such as polymer characterization,^17^ including the high-throughput measurement of polymer blend phase behaviour;^18^ but also to the fields of engineering and physics, with examples of high-throughput measurements of mechanical properties *via* micro-scale experiments^19^ and the very recently reported synthesis of multimetallic nanoclusters.^20^ In biochemistry, combinatorial libraries are usually exploited in microfluidic setups^21^ targeting cell-based applications,^22,23^ drug discovery^24^ and toxicology screening,^25^ amongst many other applications.

In most cases, high-throughput experimentation relies on the efficient and rapid generation of combinatorial parametric libraries coupled to fast characterization tools with the aim of unravelling complex multivariate spaces at high exploratory paces. This objective must be achieved guaranteeing a minimal investment on human (workforce) time and resources, while leading to a rich density of data points in the targeted space for robust conclusions. As a result, with the same amount of time and resources, high-throughput methods may explore a much greater number of systems, enabling truly combinatorial screening of donor:acceptor pairs,^26^ or making systematic studies of *e.g.* molecular weight, side chains *etc.*, with statistical relevance. Moreover, larger fractions of the parameter landscape of the systems under study can also be accessed through these methodologies. This opens the possibility to explore the parameter space much more thoroughly, and thus find the absolute maximum, or the existence of several maxima^27^ as well as critical behaviours (singularities would otherwise remain undetected).

Such unprecedented pace for data generation enables constructing large and well-structured datasets (*big data*) where machine-learning (ML) algorithms are found to have an ideal application niche. ML is thought to help in rationalizing the scientific findings, helping in the design and discovery of novel materials and orchestrating the autonomous experimentation in robotized high-throughput laboratories.

This review offers an account of the still nascent field of high-throughput screening in OPVs, with an emphasis on the synergies between computational and lab-based approximations. We first summarize some of the most relevant efforts regarding computational screening of OPV materials. Then, we describe the different existing workflows for high-throughput experimentation in OPVs, namely, the use of libraries based on gradients in the parameters of interest, schemes based on robotized labs, as well as the reported examples of self-driven labs and design of experiments. We finish by providing a comprehensive summary of the use of ML algorithms in OPVs, as well as some perspectives on the future evolution of the field.

### Definition of main concepts in the high-throughput scene

This section is devoted to introducing useful definitions of the main concepts recurrently employed in the fields of parametric optimization and experimental planning.^28^

– The term *Edisonian experimentation* refers to an experimental planning strategy in which one parameter is screened at a time with the purpose of identifying its relative performance maxima or minima based on certain figures-of-merit. For instance, one looks first at the optimum donor:acceptor ratio in an OPV blend fixing all other parameters, and from this selects the best performing composition by looking at the PCE distribution; afterwards, one fixes the donor:acceptor ratio in the previously found optimum and looks solely at the optimum annealing temperature. As depicted in Fig. 4, this approach would consist of making subsequent one-dimensional trajectories in the corresponding multivariate performance landscape with the inherent risk of bypassing the absolute maximum.

– The term *high-throughput* deals with the systematic variation of a parameter to explore its corresponding performance landscape at paces unreachable by traditional Edisonian sampling methods. These are inherently fast, which enables many parameters or/and many materials to be explored in the same amount of time as conventional methods would test just one.

– The term *combinatorial* refers to the realization of experiments in which certain material properties or recipe features are combined to change the nature of the parameters screened,^29^ so that the exploratory process turns multidimensional. In other words, *combinatorial* implies screening more than one parameter at a time with the ultimate goal of finding the absolute maximum in performance. Fig. 4 provides two examples of combinatorial evaluation, one using discrete samples (fabrication-intensive) and the other one using samples with gradients in two parameters of interest (measuring-intensive).

– The terms *optimization* and *screening* differ regarding the dimensionality of the problem. *Optimization* refers to the search of the performance maximum based on certain target function or figure-of-merit for a single system (*e.g.* PCE optimization of a particular donor:acceptor blend), whereas *screening* aims at probing uncharted parametric combinations in a more systematic manner to look for further maxima (*e.g.* screening of several acceptor materials for a given donor).

– *Computational screening* deals with performing massive quantum chemical calculations in supercomputing architectures with the objective of identifying and preselecting the most promising molecular candidates, possibly rationalizing the design and discovery of novel materials. These calculations can be run *ab initio* or biased by human intuition, *e.g.* including a bias on the ease of synthesis.

– The concept of *design of experiments* (DoE) comprises an experimental planning strategy for complex multivariate spaces that aims at discovering the overall best performing optima by executing a rationale and factorial sampling of the parametric space. In other words, DoE can be described as an experimental execution strategy that minimizes experimental effort at maximal information output.^29^

– The term *artificial intelligence* (AI) embraces a toolbox of *intelligent* computational-based methods that are able to learn from the inputs of an environment and accordingly take actions on that precise environment.^30^ Within the broad field of AI, *machine-learning* (ML) refers to all those algorithms which confer computers the ability to learn without being explicitly programmed. When these algorithms incorporate perceptrons or neural networks, they are classified as *deep learning* (DL) methods. In overall, these are able to extract correlations, trends and models from large experimental datasets or *big data* through mathematical and statistical algorithms.

– *Molecular descriptors* are useful parameters, usually in the form of numbers, that result from a logic and mathematical operation performed using the symbolic representation of a molecule or the result of some standardized experiments/calculations.^31^ Typically, these are exploited to draw quantitative structure–property relationships (QSPRs) or ML models.

– Finally, *self-driven laboratories* leverage high-throughput combinatorial experimentation and AI by combining both processes in a closed-loop pipeline. They are able to take independent decisions and execute autonomous experimentation (*i.e.* device manufacturing and characterization) until matching certain targets, thus accelerating material discovery.

## Computational toolbox for OPV materials (pre)screening

High-throughput computational studies in materials science represent an emerging field that leverages supercomputing architectures to guide the discovery of novel materials.^32^ In OPV, computational tools are designed to prescreen millions of molecular motifs and select the most promising photovoltaic candidates based on distinct *descriptors* extracted from quantum chemical calculations, such as HOMO/LUMO energy levels,^33^ oscillator strengths,^34^ absorption spectra or exciton diffusion lengths;^35^ as well as macroscopic figures-of-merit, such as the PCE, according to simplified device models (*e.g.* Scharber model).^36^ Furthermore, descriptors based on molecular graphs (which represent connection paths of atoms in a molecule) are exploited in conjunction with *generative models* and *genetic algorithms*^33^ to propose new materials based on the best-performing molecular motifs including as well a bias on the ease of synthesis.^37^ A thorough mining of molecules and the application of ML algorithms will eventually result in the establishment of predictive models based on such descriptors, but also in the rationalization of some of the findings, as these can serve to identify key molecular fingerprints to guide further materials synthesis and device prototyping. Finally, the computational forecasts can be calibrated using experimental data taken from literature or public repositories to improve the reliability of the calculations (*vide infra*).

The most extended high-throughput *in silico* screening study in OPV comes from the Harvard Clean Energy Project (CEP),^38^ which represents the first example of computational virtual screening of molecules with the aim of understanding the structure–property relations in OPV-related materials. The outcomes of the CEP have been evolving since its presentation and launch as a volunteer-driven interconnected calculation platform through the IBM World Community Grid back in 2017. The CEP was initially exploited to screen up to 1.3 million donor materials, as obtained from the combinatorial bonding of 26 different molecular fragments extracted from the literature. A more recent implementation of the CEP algorithm includes generational models biased by the human intuition^39^ to build a combinatorial library of molecules with potential interest in OPV, thus limiting the exploration space of the screening routine. Interestingly, the fundamental parameters retrieved from the *ab initio* quantum chemical calculations are publicly available and stored in a repository.^40^ Some of these datasets, namely the HOPV15 dataset, have already been exploited in training ML models and predicting key properties of OPV devices, such as PCE.^41^

**Fig. 3:**
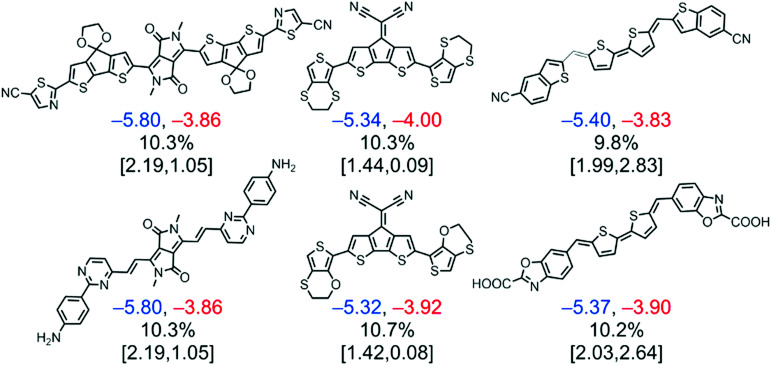
A computational screening on NFA candidates with experimentally GPR-based calibration resulted in a selection of top molecules from the quinoidal thiophene, dicyanocylopentadienyl, and diketopyrrolopyrrole fragments with predicted PCEs >9%. Under each molecule, three lines of calibrated and computed values are reported. The calibrated HOMO (blue) and LUMO (red) energies are given in the first row and are reported in eV. The second row shows the PCE based on calibrated HOMO and LUMO energies. The third row gives the computed S_0_ → S_1_ transition energy (reported in eV) and oscillator strength computed using time-dependent DFT. Reprinted from ref. 34, Copyright 2017, with permission from Elsevier.

More recently, a library of 51 000 potential NFA materials was systematically screened as a result of the combination of 107 different molecular fragments including cores, spacers and terminal groups.^34^ In this work, the lowest energy conformers of the molecules are geometrically optimized *via* density functional theory (DFT) to obtain their corresponding HOMO and LUMO energies, which are then calibrated against experimental values using a Gaussian process regression (GPR) based on their molecular similarity. These are finally exploited to estimate the PCE of the corresponding blends by means of the Scharber model,^36^ which is calibrated as well following GPR. Furthermore, a statistical fingerprint analysis shows fragment ligation patterns that increase the absorption properties of the molecules in the visible range of the solar spectrum, such as those including diketopyrrolopyrrole and quinoidal thiophene moieties (Fig. 3). However, models including the microscopic features of these materials in OPV devices are still elusive, thus the obtainment of proper correlations between the theoretically screened and experimentally observed PCE might be faulty in many cases. Also, the role of the side chains is omitted in computational screening studies due to their inherent increase in computational cost; conversely, side chains play a critical role in determining the solid-state microstructure attained in devices and also in diluting the observed oscillator strength,^42^ as these are typically saturated backbones showing poor optical absorption in the spectral range of interest.

**Fig. 4:**
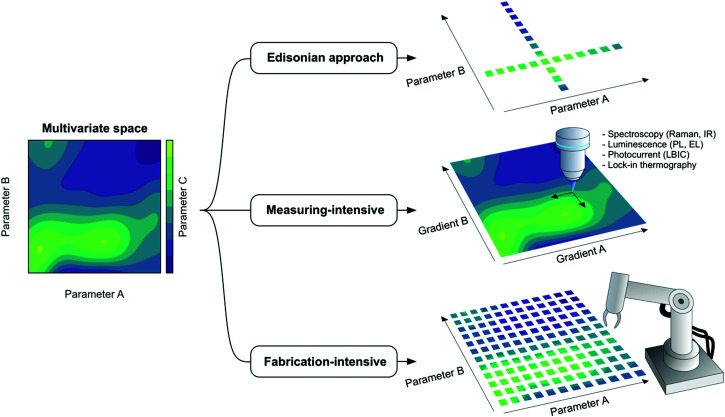
In multivariate spaces of two variables (parameters A and B), the target feature (parameter C) generally shows a complex multimodal distribution of peaks and valleys that requires careful evaluation, especially if A and B are correlated variables. In OPV, this problem is typically encountered in the optimization of active layer features such as thickness and composition (donor:acceptor ratio), which primarily determine the device performance. The classical Edisonian approach (or one-variable-at-a-time) starts by scanning A for a fixed value of B, followed by scanning of B while fixing A to the optimum value previously found. In uncorrelated systems, such strategy will generally lead to the absolute performance maximum yet in correlated parametric landscapes the conclusions might be faulty. Accordingly, at least two distinct high-throughput experimentation routines have been introduced so far to tackle the screening process in a more efficient manner while revealing the true absolute maximum: those based on lateral gradients on the parameters of interest, which rely on the intensive measuring of the target property by optical probes; and those based on the intensive prototyping of devices in automated setups (robotic arms). The latter is amenable to be orchestrated in closed-loop pipelines including DoE and self-driven labs with the help of AI.

Apart from quantum chemical calculations, high-throughput screening of other device properties, such as degree of transparency and PCE in non-opaque photovoltaic modules have also been demonstrated, reaching efficiencies close to 11% with a visible-light transparency of 30%.^43^ In a related work, other authors have used multiple optical simulations to define the best material strategies to tune colour in photovoltaic devices.^44^ Interestingly, there exist freely available software to perform both optical and electrical simulations of OPVs, such as the *gpvdm* code developed by R. MacKenzie.^45^

From the commercial viability standpoint, factors such as lifetime and cost are as relevant as device performance, thus they also have to be considered as weighting factors in upcoming high-throughput computational screening studies. These needs motivated the introduction of a metric that summarizes all three key factors (*i.e.* performance, stability and cost) in the so-called *industrial figure-of-merit* (i-FOM), as termed by C. J. Brabec's group.^46^ Similarly, Po *et al.* explored the *synthetic complexity* (SC) of active layer donor polymers^47^ and NFAs,^48^ while Moser *et al.* introduced a simplified metric termed as *scalability factor* (SF) accounting for the semiconductor synthetic cost.^49^ Based on our cost analysis, close to 50% of the overall device cost is solely ascribed to the obtainment of the raw semiconducting materials (see Table S1 in the ESI†), which highlights the importance of identifying both high performing and ‘*click-synthetized’* molecular candidates. In general, the device cost is proportional to the total number of synthetic steps required by each of the organic semiconductors employed, which is affected as well by their synthetic yield.^50^ In this regard, the donor polymer known as PTQ10 has emerged as a very cost-effective counterpart for upscaling due to its great balance between synthetic cost and performance (PCE/SF = 0.53) when blended with NFAs from the Y-series.^49^ On the other hand, state-of-the-art low bandgap polymers including PBDB-T and their halogenated derivatives do not exceed the PCE/SF threshold of 0.4. According to this report, ternary blends including low SF semiconductors (such as P3HT) are prominent alternatives from the economic perspective since they have as high PCE/SF values as the PTQ10-based devices. Therefore, a more sophisticated computational pre-screening of OPV semiconductors should look for potential candidates weighted by scalable figures-of-merit and ease of synthesis.

To overcome this issue efficiently, AI and organic chemistry are becoming jointly orchestrated in the field of computer-aided synthesis planning.^51^ Coley *et al.* deployed a ML model that optimizes and accelerates the search of organic target molecules using a database with more than 15 000 experimental reaction records. The procedure is assisted through a learned synthetic complexity metric (like SF) while simplifying the computer-suggested synthetic pathway.^51^ Yet being so far solely applied to small molecules (*i.e.* drugs and pharmaceutical compounds alike),^52^ the extrapolation of computer-aided synthesis planning to distinct fields in materials science is potentially subversive.^53^

## High-throughput experimentation workflows in OPV

High-throughput experimentation approaches in OPV can be classified into two main branches depending on the actual density of the parametric libraries that are generated, as inspired by the properties of the *analog* and *digital* worlds (Fig. 4). Measuring-intensive approaches comprise the generation of continuous (*analog*) parametric gradients whereas fabrication-intensive workflows rely on the automated fabrication of prototypes by robotic arms in discrete (*digital*) steps. Gradients intrinsically require the development of fast and high-resolution characterization and data acquisition platforms to spatially resolve the lateral parametric variations and correlate them with accessible device properties such as the photocurrent. On the other hand, robotized setups are intrinsically more compatible with standard characterization setups yet offering by default a larger consumption of raw materials and the use of highly specialized fabrication equipment (robotic arms and sophisticated control loops). In this section, we detail the latest advances and some of the still untackled bottlenecks of high-throughput manufacturing frameworks in OPV.

### Solution-processed high-throughput and combinatorial libraries

Lateral gradients comprise a continuous variation of certain parameter(s) spanning from few mm to several cm in space. In thin film technologies, the most extended application niche of parametric gradients is represented by the film thickness. Thickness gradients are of recurrent use since distinct optical interference phenomena can be discerned using such type of *analog* architectures with minimal experimental effort. Thickness gradients serve to locate the optimal active layer thickness that maximizes light harvesting in organic solar cells^54^ and to tune on demand the resonant wavelength in optical cavities.^55^ However, lateral gradients in thin films are not restricted to thickness and might include other features such as the blend ratio in multi-component mixtures or the annealing temperature, amongst others. Herein, we discuss the different experimental approaches developed so far to generate such thin film lateral gradients in a controlled manner while showing some of their unique applications in high-throughput OPV screening.

For the last 20 years of research in organic thin film libraries, blade coating and similar meniscus-guided^56^ coating techniques (such as slot-die coating)^57^ have been widely employed in the realization of continuous lateral gradients from solution with outstanding results and thus we focus here on these. Examples of alternative methods will also be mentioned.

**Fig. 5:**
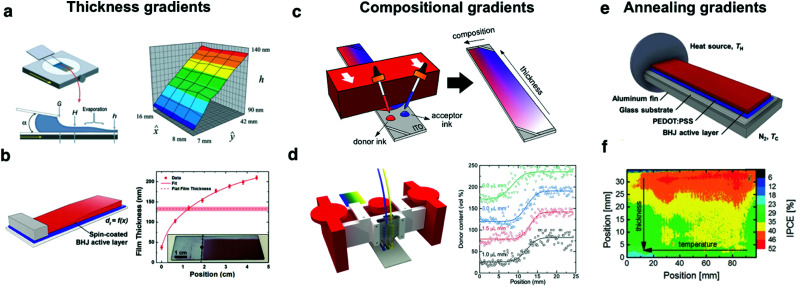
Combinatorial libraries in the form of lateral gradients can be generated following a myriad of methods while covering distinct OPV device features. Thickness gradients can be generated by (a) meniscus-guided coating techniques such as flow coating;^60^ or (b) off-center spin coating.^64^ Compositional gradients, where distinct donor and acceptor inks are blended in a controlled fashion, can be realized by (c) a two-drop coalescence method based on blade coating;^54^ or (d) using a microfluidic-assisted blade coating platform that assures enhanced reproducibility.^70^ Annealing gradients serve to screen how the microstructure of the photoactive layer affects device performance and these are typically realized by (e) holding one side of the sample at a fixed temperature *T*_H_, thus generating a continuously decreasing temperature profile (up to *T*_C_) spanning over the long aspect ratio substrate.^64^ (f) Annealing gradients are usually combined orthogonally with gradients covering a distinct parameter such as thickness, thus realizing 2D combinatorial libraries.^54^ Figures reprinted from ref. 60, with the permission of AIP Publishing; ref. 64 (Copyright 2017, American Chemical Society); and the authors.^54,70^

Thickness gradients constitute one of the simplest high-throughput libraries that can be readily produced by blade coating. Linear thickness gradients spanning from a few tens of nm to several hundreds of nm in a single sample have been demonstrated in the Landau-Levich lubrication regime.^54,58,59^ In these conditions the wet film thickness depends mainly on the coating speed; therefore, a continuous thickness variation in solid-state is achieved by employing a blade applicator actuated at constant acceleration, rather than at constant speed (Fig. 5a).^60^ Blade coated thickness gradients can span over large areas with excellent homogeneity (*ca.* 20 cm^2^ have been demonstrated),^54,61,62^ rendering them useful to explore interference phenomena in optoelectronic devices in a high-throughput manner. These gradients were accordingly employed to tailor the optimal active layer thickness in bulk heterojunction, polymer:small molecule solar cells;^63^ tandem solar cells with their active layers arranged in an orthogonal fashion;^62^ and to accelerate the screening of the thickness dependence on the photovoltaic performance and stability.^61^

While blade coating is probably the most controllable and material-efficient technique to produce thickness gradients, other approaches have been used and are worth mentioning. Interestingly, there is one literature report demonstrating off-center spin coating to generate lateral film thickness gradients in P3HT:PCBM blends with good reproducibility (Fig. 5b).^64^ While in spin-coating a significant fraction (>90%)‡‡For a 10 mg mL^−1^ solution and 1 cm^2^ substrates, spin coating at least requires depositing a drop of 50 μL (or 500 μg) to homogeneously cast a film. Assuming that the spin coating procedure leads to a 100 nm-thick film with a density of 1 g cm^−3^, this results in 10 μg dried-deposited on the film, or 98% of the original solid content wasted. These are conservative numbers, as by spin coating typical concentrations are closer to 20 mg mL^−1^ and 100 μL, which would lead to 2 mg of material first deposited and 99% of waste. Similarly, when employing 4 cm^2^ substrates with 10 mg mL^−1^ solutions and a 50 μL drop, the percentage of solid content wasted lowers to 92%. of the deposited ink is spun out of the substrate, and thus is not an optimal method for high-throughput screening, the fact that it is so widely available may render the technique useful. On the other hand, using movable shadow masks, evaporated devices with thickness gradients can also be obtained.^65^ Recently, evaporated OPVs with bilayer architecture have been optimized by using orthogonal wedges of donor and acceptor layers.^66^ In addition to lab-scale research, solution-processed thickness gradients have also been demonstrated to be an efficient way to screen the active layer thickness dependence in roll-to-roll (R2R) setups. Alstrup *et al.* fabricated flexible solar modules including controlled thickness variations along the web by adjusting the flow rate fed in slot-die coating heads.^57^

The generation of combinatorial compositional libraries in solid-state is certainly more experimentally challenging yet it renders attractive in those systems in which the mixing ratio strongly influences the device performance, such as the case of OPV and the donor:acceptor ratio. Since composition affects the amount of light harvested and the efficiency of exciton splitting and charge collection, every novel donor:acceptor pair must be subjected to its corresponding optimization. In 2010, F. C. Krebs's group implemented a differentially pumped slot-die coater in a R2R setup to realize a one-dimensional compositional library as an elegant way to accelerate the donor:acceptor ratio optimization process in OPV devices, which they demonstrated for the P3HT:PCBM system.^57^ Two years later, Lee *et al.* reported the fabrication of lateral compositional gradients using spray-assisted deposition methods although showing very limited intermixing of the donor and acceptor materials.^67^ Since then, and given the unsparingly growing catalogue of light-harvesting organic semiconductors, different high-throughput experimental approaches have been developed to be compatible as well with lab-scale research laboratories.

Current envisioned approaches to process compositional gradients in solid-state directly from solution merge previous knowledge and ideas on liquid-based gradient generation (including also microfluidic arrays) with meniscus-guided coating techniques such as blade coating or slot-die coating. Compositional libraries in solution were realized in the early century by the prototyping of microfluidic branched arrays containing long zig-zag streams.^68,69^ These were demonstrated to enable a fine-tuning of the mixing profile obtained at the outlet, yet their actual transfer to solid-state film profiles was demonstrated more recently as a result of a synergic combination of microfluidic devices and blade coating (Fig. 5d).^70^ In this methodology, 3D-printed microfluidic devices were used to control the *in situ* mixing of the pristine donor and acceptor inks, which are poured directly as three independent streams (including a central branch with a 1 : 1 (v : v) mixture) to the blade ink reservoir during the coating process. Then, the movement of the applicator drives their coalescence at the wetting edge, resulting in smooth compositional profiles in solid-state for polymer:small molecule blends as well as the more challenging all-polymer counterparts. Importantly, less than 50 μL of ink per material are required to perform the experiments, while the original ink vials remain unaltered as well for further experimentation.

In the particular case of polymer:small molecule binary blends, the rheological characteristics of the inks are not as demanding and simpler, yet less controllable processing schemes are possible to realize combinatorial libraries. These were first demonstrated by Sánchez-Díaz *et al.* for binary bulk heterojunction blends comprising a donor polymer and a fullerene acceptor (Fig. 5c).^54^ In this approach, neat ink drops of the materials under study are cast with a multichannel micropipette adjacently at the blade reservoir. Afterwards, the movement of the blade coater smoothly merges both inks generating a compositional gradient approximately perpendicularly to the blade direction, a feature which opens the possibility to manufacture as well an additional thickness gradient (2D gradients) along the coating direction by accelerating the applicator. Very recently, our group applied the same methodology to heterogeneous blends of donor polymers and NFAs.^26^ The possibility to pattern 2D gradients over large areas is unique to blade coating, thus advancing the previous 1D exploration based on differentially-pumped slot-die coating.^57^

Ternary OPV blends, which offer a series of long-term stability and performance advantages over the classical binary bulk heterojunctions,^71^ have also been effectively screened by means of compositional libraries realized by blade coating.^27,72^ In this case, a layer-by-layer strategy was employed to subsequently deposit, one on top of the other, the different layers of the constituent active layer materials. Depending on the layer ordering and velocity profile employed (either constant speed or acceleration), complementary areas of the ternary diagram are covered, so that less than 5 combinatorial samples are required to explore a significant fraction of the ternary phase diagram.

Temperature or annealing gradients are performed on dedicated heating stages such as Kofler benches, which generate controlled linear temperature variations over a long plate spanning up to a few hundreds of °C over several cm (Fig. 5e). Pascual-San-José *et al.* employed 1D thermal gradients to determine the optimum annealing temperature in P3HT:NFA based devices.^61^ In the most frequent arrangement, the temperature gradient is oriented perpendicularly to a secondary gradient such as film thickness, thus generating 2D combinatorial libraries in a single substrate (Fig. 5f).^54,64^ These are useful to leverage the exploration rate of multi-dimensional parametric spaces such as phase diagrams^73^ or the relationship between device performance and active layer film morphology in OPV devices.^54,61,64^ Other examples of 2D combinatorial libraries embrace active layer thickness and compositional libraries, which have been recently exploited to perform the high-throughput combinatorial screening of OPV blends with minimal time and resources investments.^54^ Besides performance, annealing gradients were recently employed to assess the relationship between post-thermal treatment and photostability of state-of-the art OPV materials.^74^

### Optoelectronic characterization of lateral thin film gradients

High-throughput experimentation based on lateral parametric gradients requires characterization techniques that are able to spatially resolve and quantify the generated libraries (material properties) while correlating them with the corresponding photovoltaic performance (device properties). These techniques are, thus, a secondary yet fundamental kingpin supporting high-throughput screening using gradients. Therefore, they should ideally be non-destructive and fast in terms of data-acquisition to leverage their unique throughput capabilities.

**Fig. 6:**
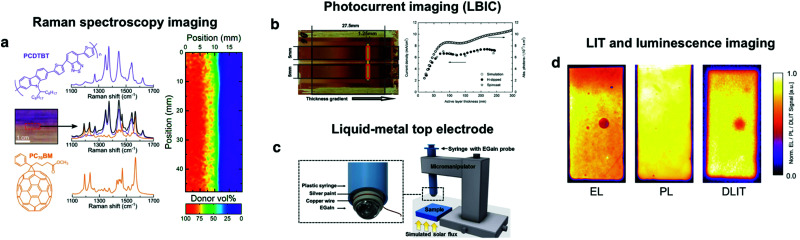
After generation of the combinatorial libraries, compatible characterization methodologies include (a) Raman spectroscopy imaging, which serves to quantify active layer thickness and composition variations over large areas;^70^ (b) photocurrent imaging, which can be performed using either a focused laser beam source^27,54,70^ or a movable shadow mask to illuminate controlled portions of the sample while extracting the corresponding *J*_sc_;^63^ (c) liquid-metal top electrodes in devices without evaporated top electrodes, having the intrinsic advantage of extracting local *JV* curves throughout the combinatorial library;^64^ and (d) a combination of dark lock-in thermography (DLIT) and luminescence imaging (including electroluminescence, EL, and photoluminescence, PL) to evaluate the presence of defects in any of the interlayers forming the OPV device stack.^76^ Figures reprinted from ref. 63, Copyright 2012, with permission from Elsevier; ref. 64 (Copyright 2017, American Chemical Society); ref. 76, with permission from John Wiley & Sons; and the authors.^70^

In this regard, optical characterization techniques represent the ubiquitous choice in OPV. This is motivated by the fact that shadowed or focused light beams can locally induce the photogeneration of charge carriers throughout the gradients, being the photocurrent an excellent proxy of the overall photovoltaic performance (as we have statistically demonstrated analyzing our in-house database of more than 5000 devices, see Fig. S1 in the ESI†). When carefully combined with motorized XY stages and automated acquisition software, light-beam induced current (LBIC) setups retrieve spatially resolved photocurrent maps that are extremely valuable for gradient-based high-throughput experimentation in OPV. In 2012, Nickel and co-workers first exploited wedge-shaped photoactive layers in combination with a mask mounted on a motorized stage to optimize the active layer thickness in polymer:fullerene blends (Fig. 6b).^63^ A few years later, the same group, led by A. Colsmann, demonstrated the high-throughput optimization of organic tandem solar cells using orthogonally oriented absorber layers.^62^ Therein, the local current density was accessed using a movable shadow mask with either a slit or a pinhole aperture. An additional probe was required to quantify the lateral film thickness variations (or other gradients alike such as composition or crystallinity) and correlate them with the photocurrent retrieved by means of LBIC mapping. Regarding film thickness, these are typically destructive techniques such as profilometry^63^ or confocal microscopy,^62^ which is still largely invasive since the determination of the thickness requires scratching and peeling off several layers from the device stack. Despite these drawbacks, the authors demonstrated a very efficient use of materials and evaluation times that were faster than conventional optimization protocols.

Recently, our group introduced a co-local optoelectronic characterization technique combining Raman spectroscopy and LBIC maps in a single confocal setup that embraces both non-destructiveness and fast data acquisition (Fig. 6a).^27,54,66,70^ Raman spectroscopy is exploited to quantify the local thickness and composition of the active layer with diffraction-limited resolution by carefully deconvoluting the measured spectra.^75^ Interestingly, these measurements are performed on a full device stack, thus the same laser beam can be exploited to extract simultaneously the corresponding photocurrent map under monochromatic excitation. Importantly, this evaluation is performed non-invasively over large areas (cm^2^) at an unbeatable pace (close to 20 000 spatial positions are scanned per hour). Beyond monochromatic light, broadband excitation has also been recently incorporated to the setup albeit doubling the measuring time required to complete the characterization (as it cannot be retrieved on-the-fly during Raman spectra acquisition). This methodology has now been extensively exploited to demonstrate the high-throughput optimization of binary^26^ and ternary^27^ OPV blends.

In addition to LBIC, other non-destructive imaging methods such as lock-in thermography (LIT) and luminescence imaging (including both electroluminescence, EL, and photoluminescence, PL) are techniques of frequent use in quality control when upscaling OPV modules (Fig. 6d).^76^ For that reason, they are natively fast evaluation techniques compatible with large area devices that keep an excellent sample preservation. When applied to lateral parametric gradients or libraries, these techniques can be leveraged as qualitative imaging tools in high-throughput experiments despite not being able to quantify their lateral distribution, such as thickness or composition (as Raman spectroscopy does). Conversely, these provide reliable and quick qualitative assessments of distinct device-scale properties such as defects, degree of homogeneity or overall device performance in fully operational devices. Likely, it is simply a matter of time for these techniques to be eventually exploited in conjunction with combinatorial parametric libraries in high-throughput experiments based on lateral gradients.

Dark lock-in thermography (DLIT) serves to visualize thermal losses in operational solar cells under electrical excitation, with the ability to resolve spatially the shunt and contact resistances.^77–79^ Conversely, illuminated lock-in thermography (ILIT) studies the temperature distribution of the devices under optical excitation,^80^ serving as well to quantify shunt resistances in OPV modules.^81^ LIT renders useful to study degradation in organic solar cells^82^ employing broadly accessible pieces of equipment such as a digital lock-in, a thermostat as sample holder and an infrared camera. Since the major contributor to the observed rise in device temperature is the large resistivity of the active layer, DLIT can be exploited as well to image its inhomogeneities and defects^77^ as demonstrated by Hoppe and co-workers.^79^ While we are not aware of its use in samples with gradients, this technique could complement LBIC as an indirect method to evaluate local series resistance, and thus, as a proxy of the FF.

Luminescence imaging is exploited to resolve the spatial distribution and quantify the efficiency of the radiative recombination processes taking place in OPV devices. PL imaging evaluates the recombination taking place in the active layer only whereas EL imaging embraces as well the quality of the charge injection and extraction of the full device stack. As a result, EL imaging can easily identify cracks or malfunctioning areas on working photovoltaic modules as being an excellent prior of the short-circuit current distribution.^83^ In this regard, Doll *et al.* introduced a camera prototype including indium gallium arsenide detectors with integration times as low as 5 ms to acquire EL images in a high-throughput fashion. Their setup is applied outdoors in photovoltaic installations under low-light conditions and even at daytime.^84^ On the other hand, PL mapping serves to evaluate the degree of phase separation between the donor and acceptor species by looking at the PL intensity quenching and shifting. Moreover, PL imaging has been exploited as a marker of the *V*_oc_ in ternary OPV libraries^27^ and to evaluate the efficacy of the laser-patterning process in scalable OPV devices.^85^

Apart from optical characterization approaches, Savagatrup *et al.* introduced the photovoltaic mapping of gradients (PVMAP) by employing a liquid metal top electrode that reversibly contacts OPV devices without evaporated electrodes (Fig. 6c).^64^ The non-damaging liquid metal is positioned using a micromanipulator to map how thickness and morphology gradients (arranged as a 2D gradient library) control performance in air-exposed P3HT:PCBM samples. This approach leverages gradients and the movable liquid-metal top electrode to extract local *JV* curves over mm^2^ areas yet having the limitation of not reproducing the exact conditions found in functional devices, where a well-defined interlayer and top electrode are included for correct operation.

### High-throughput experimentation in robotized laboratories

In the second half of the 20th century, the substitution of the human workforce by robot arms resulted in major breakthroughs in the industrial production models worldwide, where fabrication paces reached unprecedented levels.^86^ Accordingly, robots are expected to keep on replacing humans in the most repetitive tasks (including those in science) due to their unbeatable yield and batch-to-batch reproducibility while leaving non-repetitive experiments, as well as the rationalization of results and proposal of groundbreaking ideas and experiments (almost) solely to researchers. These features render robots particularly profitable in those research scenarios designed as a discretized (*digital*) exploration of multi-dimensional parametric spaces. Following these guidelines, automated experimentation constitutes an emerging field in materials science that inherits most of the previous know-how from the high-throughput pharmaceutical industry^87^ and combinatorial chemical synthesis.^88,89^ In the latter, the use of robots extends for several decades now, with the first example of closed-loop automated chemical synthesis dating from 1978.^90^

**Fig. 7:**
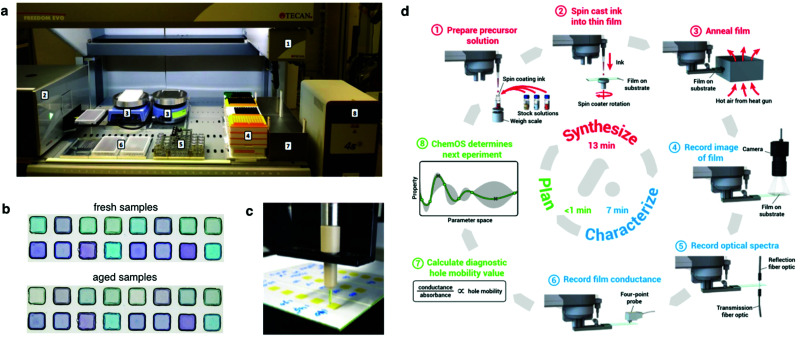
Setup and outcomes of robotized laboratories. (a) Front view of a semi-automatic robot system consisting of: (1) robot arm with four pipetting channels; (2) spectrometer; (3) two hot plates; (4) different sizes of tips; (5) stock solutions for experiment; (6) 96-well microplates; (7) waste container; and (8) heat sealer.^93^ (b) Outcomes of a high-throughput experimental study performed in quaternary OPV blends to assess stability against continuous illumination. In this case, samples were prepared in the robotized laboratory shown in (a) by drop casting.^93^ (c) Inkjet printed array of ternary OPV blends for their combinatorial optimization in terms of extended light harvesting.^95^ (d) Representative workflow in a self-driven laboratory that merges automatic ink preparation and film casting, optoelectronic characterization and experiment planning in a closed-loop pipeline with the help of ChemOS and AI.^110^ Figures reprinted with permission from ref. 95 (Copyright 2013, American Chemical Society); and the authors.^93,110^

In photovoltaics, modern robotized laboratories aim at gathering sample fabrication and basic characterization (such as *JV* curve extraction) in a specifically designed modular setup, including robotic manipulation, custom electronics and dedicated sample holders (Fig. 7a).^91^ This results in highly specialized production lines with a somehow limited adaptability to applications out of their original scope. It enables, however, an efficient exploration of the parametric space. From a conceptual point of view, the targeted samples are the full device stack and the characterization would be, at least, the *J*_sc_, the FF and the *V*_oc_, *i.e.* the parameters that one would extract measuring the *JV* curve under 1 sun illumination conditions. In other words, the assembly line would reproduce what is done conventionally by researchers in the lab. Although this is the starting point, automatization may open additional avenues, as we will discuss below.

In the field of organic electronics, Kiy *et al.* pioneered the implementation of a robotized laboratory for the high-throughput optimization of the luminance efficiency in polymer LEDs.^92^ Their setup was able to spin cast both the buffer layer and the emitter layer (yet not to automatically evaporate the aluminum cathode) and electrically measure up to 49 fully functional devices in 4 hours (excluding sample cleaning and data analysis steps), thus leading to a screening rate close to 5 minutes per sample. This figure is very similar to that attained in more modern robotized manufacturing setups,^93^ thus may indicate the existence of an intrinsic limitation in the automatization of thin-film fabrication protocols. One important bottleneck of this first automated approach in organic electronics lies in the spin casting process since a single spin coating already takes on the order of 1 minute, and several layers are deposited per device (active and transport layers). Moreover, at that particular stage more than 90% of the initially pipetted ink is wasted.

To partly circumvent this limitation, Teichler *et al.* introduced a platform based on inkjet printing to demonstrate the combinatorial screening of polymer:fullerene^94^ and polymer:polymer:fullerene^95^ blends (Fig. 7c). Inkjet printing offers a significantly improved material yield yet being technically challenging to implement due to the potential nozzle clogging associated with small solvent volumes, as well as Marangoni flows (coffee ring effect) when employing suboptimal inks in terms of rheology. Also, the screening realized by inkjet printing was limited to pristine films only, thus it was not possible to assess photovoltaic parameters directly at that stage; instead, Teichler and co-workers characterized and optimized the film quality based on the extension of their optical absorption, PL quenching and film smoothness.

More recently, Langner *et al.* developed an automated (and eventually self-driven) experimentation setup to evaluate the effect that small amounts of additives have in increasing the long-term stability and lifetime of quaternary high performing OPV blends.^93^ In this study, inks were drop-casted to form films, and the photodegradation was evaluated by looking at the evolution of the absorption spectra under continuous illumination (Fig. 7b). Their approach can screen up to 288 samples per day (5 minutes per sample) yet it is constrained to drop-cast films, thus bypassing the manufacture of full device stacks to extract reliable priors of the photovoltaic performance. A direct evaluation of stability may require, however, the use of full device stacks since, often, interfacial effects or ion migration from neighbouring layers might have significant effects on the final stability.^96,97^ Nevertheless, the use of low concentrated inks (0.6 mg mL^−1^), which are compatible only with drop-cast films, reduces the use of raw semiconducting materials to 15 μg per sample. For a typical high-throughput automated exploration of 500–1000 samples, the total amount of raw material required is close to 10–15 mg, which are truly competitive figures and similar to those attained using lateral gradients (see Table 1). Conversely, the large indium tin oxide (ITO) substrate consumption is a major drawback of this process since they represent close to 25% of the device manufacturing cost (see Table S1 in the ESI†).

**Table 1:** Comparative figures in terms of time and raw semiconducting material requirements in benchmark high-throughput experimentation procedures for OPV screening and optimization

Parameter	Discrete sampling	Design-of-experiments^e^	Automated experimentation			Self-driven laboratory	1D discrete gradients	2D continuous gradients
Ref.	26	112	92	93	99	93	26	26
Problem dimensionality	2D	4D	1D	4D	10D	4D	2D	2D
Casting procedure	Spin/blade coating	Spin coating	Spin coating	Drop casting	Spin coating	Drop casting	Blade coating	Blade coating
No. samples	66^a^	235 (29 steps)	49	288	100	30	11^b^	1
No. non-equivalent data points per sample	1	1	1	1	1	1	12	24000
No. data points (total)	66	235 (29 steps)	49	288	100	30	132	24000
Manufacturing time (h)	93.2^c^	328.7 (41.8)^c^	—	—	—	—	16.5	5.9
Measuring time (h)	0.55^d^	1.96 (0.24)^d^	—	—	—	—	1.12^c^	9
Data analysis (h)	1	1	—	—	—	—	2	13
Total time (h)	94.8	331.7 (43.04)	4	24	24	2.5	19.6	27.9
mg of semiconductor	79.2	470 (58)	41.6	4.3	50	0.45	13.2	1.2
Time required per data point (min)	86.4	84.6 (88.8)	5	5	14.4	5	9	1.4
μg of semiconductors required per data point	1200	2000	850	15	500	15	100	0.05
Hardware investment	Low	Low	High			High	Moderate	High
Software investment	Low	Moderate	Moderate			High	Moderate	High

a From, to neat films in 10 vol% steps + 6 homogeneous thickness steps.

b From, to neat films in 10 vol% steps + 12 thickness steps as gradient.

c Assuming 11 batches with co-evaporation of 6 samples per batch.

d *JV* curve takes 30 s to be measured.

e The values in brackets correspond to the preparation of a single replicate per optimization step (29 steps in total).

The same group led by C. J. Brabec and J. Hauch recently deployed an automated solar cell fabrication and characterization platform labeled as AMANDA Line One (Autonomous Materials and Device Application Platform, www.amanda-platform.com).^98^ In a first demonstration, they evaluated the efficiency and photostability of the workhorse donor:acceptor OPV blend PM6:Y6.^99^ The 10-dimensional multivariate space therein screened required 24 hours to be completed, and close to 72 hours including a 50 h photostability test performed with the help of Gaussian Process Regression (GPR) algorithms to identify the key process parameters. GPR predictions of photovoltaic parameters were drawn as well by incorporating an in-line measurement of absorption and its spectral decomposition, which further guides the decision-making and, thus, the screening procedure.

Apart from the realization of high-throughput screening studies on performance and stability in organic solar cells, robotized laboratories are probing an increased niche of applications in the organic electronics community. C. J. Brabec and co-workers adapted their robotized equipment to demonstrate first the systematic screening of eco-friendly nanoparticle inks for organic solar cells.^100^ In an ulterior work, the same group provided an improved understanding and modelling of the solvent-antisolvent crystallization approach typically employed to obtain high quality perovskite layers for photovoltaics.^101^ Last year, Li *et al.* performed more than 8000 metal halide perovskite synthesis reactions in an automated high-throughput approach based on inverse temperature crystallization, to then apply ML methods and retrieve predictive models for crystal formation.^102^

### Self-driven laboratories

In addition to the high yield and reproducibility, screening procedures based on massively automated prototyping offer an additional *potential* advantage: autonomous experimentation. Robotized laboratories can be orchestrated on-the-fly by ML algorithms to rationally reduce the number of samples required to perform the exploration of large parametric spaces, forming the so-called self-driven laboratories.^53,103,104^ These are especially useful in problems presenting a large interconnectivity of the screened variables, such as in OPV, since an AI-driven, closed-loop pipeline guarantees a fully autonomous execution of high-throughput combinatorial experimentation in the context of materials discovery^105^ and device optimization. These smartly automated systems aim at combining robots for the realization of the experimental procedures with search algorithms to drive their smarter use, paying close attention to the reduction of experimental samples screened to optimize the target function. The search algorithms, either ML models or Bayesian optimization approaches,^106,107^ are typically executed in-line with the experimentation, thus creating an integrated pipeline that automatically evaluates the experimental outcomes, retrains the models and proposes the remaining exploration steps. In this way, the laboratory becomes autonomous and is able to significantly reduce the consumption of raw materials, thus accelerating device optimization and materials discovery. Following such synergy between robots and AI, Burger *et al.* recently tackled ten-variable experimental spaces in their search of photocatalysts for hydrogen production from water.^106^

In the OPV scene, Langner *et al.* incorporated a Bayesian optimization algorithm to autonomously drive their robotized experimental procedure focused on stability and degradation of quaternary OPV blends.^93^ Accordingly, they demonstrated a substantial reduction on the number of data points required to complete the screening study (from 500–1000 to 30 samples only) by making use of the ChemOS orchestrating software package.^108,109^ The raw material requirements were also downscaled proportionally: from 10–15 mg following conventionally-automated high-throughput experimentation to less than 1 mg per compound in the best self-driven scenario. While very promising, their high-throughput experimental screening procedure is thus far limited to drop cast films only, hence the assessment of the photovoltaic performance itself is not encompassed in their study and it is restricted to stability evaluations on bare films.

Similarly, MacLeod *et al.* optimized the hole mobility of amorphous spiro-OMeTAD using a flexible and modular self-driving laboratory known as *Ada*, which incorporates ChemOS as controlling software^108^ and Phoenics as Bayesian optimization algorithm.^110^ Their approach includes as well the fabrication of samples by spin coating, their thermal annealing and optical characterization in terms of reflection and transmission *via* UV-vis-NIR spectroscopy and four-point probe conductance measurements to get a proxy of the pseudomobility (Fig. 7d). In this case, the time spent in the fabrication and characterization in terms of absorbance and conductance is close to 50 minutes per sample, including time for restocking consumables. In this way, they explore the effect that two variables (annealing time and dopant concentration) have on the pseudomobility fabricating only 35 samples in less than 30 hours, with great reproducibility in a fully autonomous and self-driven lab.

### Design of experiments (DoE)

Without the degree of sophistication required to perform self-driven experimentation with AI, a smart design of experiments (DoE) can be exploited to significantly reduce the number of samples needed to explore complex multivariate spaces.^111^ These strategies apply to both automated and conventional experimentation approaches and they essentially aim at avoiding to perform a full-grid search of the parameter space in individual step sizes for each of the parameters considered. In fact, DoE gathers all those planning strategies focused on *minimizing experimental effort at maximal information output*.^29^

The application of DoE strategies such as factorial design in OPV was first introduced by J. M. Buriak's group, who replaced Edisonian experimentation by a statistically-based rationale in the selection of devices to be fabricated while optimizing the use of human workforce time and resources.^112^ In a first optimization round of their PCDTBT:PC_71_BM solar cells, a four-variable parametric space consisting of donor weight ratio, solution concentration, spin speed and concentration of processing additive is screened according to a Latin square factorial sampling approach followed by an analysis of variance (ANOVA). Other variables such as the temperature and duration of the annealing treatments have been recently optimized as well for NFA-based devices following the same analysis.^113^ In this first round,^112^ one of the parameters is dropped (presence of additive) and the remaining data is fitted using a ML algorithm (a support vector machine (SVM) model regressor) to obtain a complex hypersurface describing the PCE. In a subsequent optimization round, the range of values experimentally screened is narrowed to fine-tune the experimental conditions that maximize the PCE. This approach renders useful to reduce the number of samples fabricated when optimizing multi-parametric spaces of correlated features in parallel, and it also increases the probability to discover the *true* optimum. However, this testbed optimization procedure for a four-dimensional space of a polymer:fullerene blend still required up to 235 samples to be prototyped (distributed in 29 different exploratory steps) due to the large number of replicates required to build robust statistics. This results in a raw material consumption close to 470 mg of semiconducting materials only (100 μL per sample at an average concentration of 20 mg mL^−1^). Therefore, the investments in terms of time, cost and raw materials might be inadequate to meet the demands of high-throughput experimentation in OPV. Likely, DoE has a broader potential to decrease time and resources requirements when applied in combination with robotized and self-driven laboratories, where the higher reproducibility of the manufacturing process could be exploited to reduce the number of replicates and control samples to a few units only. Still, factorial design is typically limited to the simultaneous optimization of five parameters at two levels in the best case scenario.^29^ Beyond that degree of experimental complexity, human intuition ought to be exploited to prescreen the parameter space. Conversely, A. Aspuru-Guzik's group has very recently introduced *Golem* in the DoE scene as a novel process and experimental optimization algorithm.^114^*Golem* is able to minimize the impact of noise in the existing experimental conditions while providing robust solutions and decision-making on-the-fly using the outcomes of past experiments.

### Throughput assessment comparison between approaches

At this stage, two main protocols for realizing high-throughput experimentation in complex multi-parametric scenarios have been described: gradients, spanning either one or two dimensions; and robotized laboratories in any of their variants, *i.e.* automated experimentation/characterization or fully self-driven laboratories. We next perform a one-to-one comparison in terms of experimental time required and raw material consumptions in each of them, in order to benchmark the distinct methodologies available nowadays. Accordingly, we summarize in Table 1 the main figures retrieved from seminal papers on each of the high-throughput experimentation approaches covered in this review, including also the classical Edisonian protocol and experimental planning strategies as per the principles of DoE. We note that the comparison only refers to already demonstrated cases, rather than the full potential of each approach.

Regarding automated experimentation, the pioneer work by Kiy and co-workers already set the figures-of-merit of automated approaches. Given the small active area (4 mm^2^) of their devices, our estimations indicate a consumption of raw active layer materials close to 40 mg to complete a 49-devices batch (*i.e.* 50 μL per device at a concentration of 17 mg mL^−1^), thus leading to a raw material consumption rate close to 0.85 mg per device (*i.e.* close to the standard figures in spin coated devices) while accessing to relevant device figures-of-merit (luminance efficiency in the case of LEDs). These numbers, despite the automatization of the manufacturing process and the unbeatable device fabrication yield, might still be prohibitively high for the exploration of complex multi-dimensional landscapes when only small synthetic batches of the raw materials are readily available. Conversely, their synergic implementation with AI algorithms in an integrated workflow can lower the consumption of materials to figures below 5 mg (and even 500 μg) in high-dimensional optimization studies.^93^ However, the access to photovoltaic figures such as the *J*_sc_ must yet to be accomplished and accordingly included in the time and material spent per experimental data point. In this regard, that same research group recently tackled a 10-dimensional parametric space including access to the photovoltaic figures-of-merit (*J*_sc_, *V*_oc_, FF and PCE) while increasing by one order of magnitude the semiconductor material requirements up to *ca.* 50 mg.^99^

Comparatively, gradient-based screening methodologies arise as one of the most cost-effective and straightforward approaches toward the fast screening of multivariate spaces. 1D gradients comprising discrete devices offer throughput figures comparable to those of state-of-the-art robotized laboratories as well as a very efficient consumption of raw materials (of a few tens of μg per data point). Furthermore, these allow measuring *JV* curves in fully operational devices, thus accessing the complete catalogue of photovoltaic figures-of-merit at each screening step. Also, the investments in terms of equipment are reasonably low as the procedure only requires an accelerated meniscus-guided coating platform such as blade coating to generate the gradient-based libraries, or even solely an spin coater.^64^ Moreover, the realization of 2D gradients comprising a single large area electrode further maximizes the throughput of this type of parametric libraries: time and materials requirements can be set below 2 min and close to 50 ng per data point in the best scenario. An important shortcoming when using combinatorial 2D devices is, however, the fact that it only accesses photocurrent as a proxy of the photovoltaic performance. Moreover, the procedure also requires a larger initial investment in specialized equipment (namely Raman spectroscopy imaging setups).

Beyond the use of raw semiconducting materials, a more detailed material cost analysis illustrates that in those cases where transparent conductive oxides such as ITO are commercially obtained and employed as substrate, it is the actual cost of the substrates what acts as limiting factor in the implementation of a cost-effective screening procedure. In particular, when employing commercially-available OPV materials, our calculations show that the cost of the ITO substrates typically covers 25 to 50% of the total price of the devices (see Table S1 in the ESI†). In these cases, high-throughput approaches that minimize the number of prototyped samples have larger associated value, which increases the potential advantages of implementing gradient-based screening procedures against other methodologies.

## The reward after high-throughput experimentation: *big data* scenarios

**Fig. 8:**
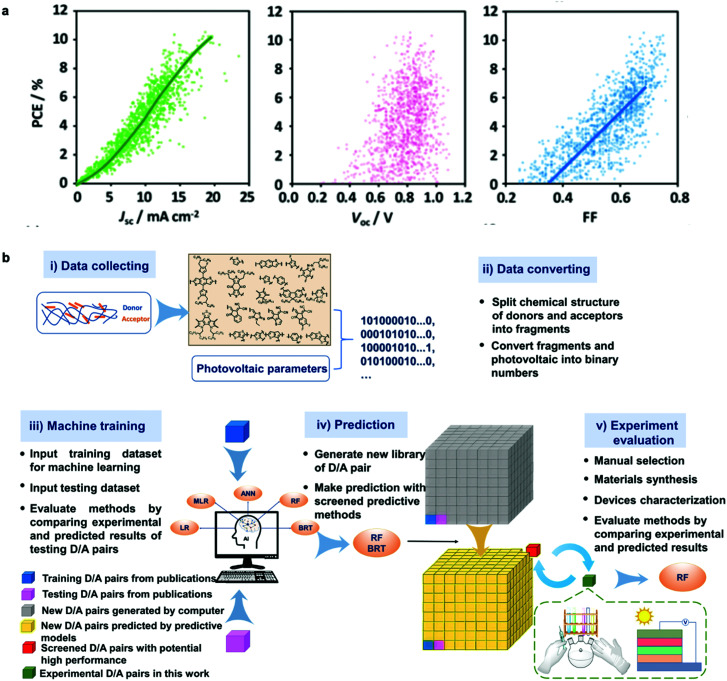
(a) Correlation between PCE and the photovoltaic figures-of-merit (*J*_sc_, *V*_oc_ and FF) as reported by Nagasawa *et al.* after manually collecting close to 1200 values from literature (*ca.* 500 articles were accessed). The data mining included HOMO–LUMO energy levels and molecular weights. The resulting database was then exploited by artificial neural networks and random forest methods to find potentially high-performing choices for polymer:fullerene OPV devices.^119^ (b) Workflow employed by Wu *et al.* for building, applying and evaluating ML methods to identify and synthesize high-performing molecular candidates for OPV applications.^131^ Images reprinted (adapted) with permission from ref. 119 (Copyright 2018, American Chemical Society); and the authors.^131^

High-throughput experimentation generates data at unreachable paces by any other classical experimental approach. Accordingly, *big data* readiness is experiencing a notorious upswing in many scientific areas in the form of publicly available repositories, including both experimental and theoretical datasets. In parallel, the capabilities to extract valuable information from large datasets have been growing exponentially since the introduction of the *knowledge discovery in databases* back in the late 80s.^115^ Nowadays, such an old-fashioned term was renamed as *data mining*, which refers to the process of extracting implicit information from data stored in databases.^116,117^ In materials science and OPV in particular, data mining procedures are showing an increased potential for material discovery through generative models in combination with ML algorithms, which are able to retrieve performance predictions by inspection of large datasets (Fig. 8b). In fact, the potential of ML algorithms for high-performing organic solar cells and data-driven discovery of new materials has been very recently reviewed in this precise journal.^118^ In this section, we present the latest advances on data mining and data repositories as well as their exploitation by means of AI algorithms to accelerate OPV materials screening and discovery.

The organization of data repositories in academic laboratories has been more elusive than in industry, where it is more common to perform a detailed tracking of the samples and experimental conditions followed in device manufacture and testing. Nevertheless, in our own research laboratory the introduction of a rigorous labeling system served us to build a thorough database of more than 5000 entries over four years of high-throughput experimentation based on 1D lateral gradients. This database was first exploited to statistically demonstrate that *J*_sc_ is the best proxy for PCE (see Fig. S1 in the ESI†), in excellent agreement with other data mining studies (Fig. 8a).^119^

However, in general the application of data mining in OPV requires performing a manual retrieval of data from literature to build a large enough database to extract statistically robust conclusions. These data are typically accessed either from the abstracts or the main text of the articles, thus constituting a highly time-consuming process performed entirely by humans due to its complexity. Therefore, authors are encouraged to submit their curated high-throughput datasets^120,121^ in conjunction with the conventional supplementary information to ease the build-up of databases.

For this reason, initiatives promoting a careful indexation of the most prominent results in public repositories are highly valuable for the development of the corresponding technology. In thin film inorganic photovoltaics, the publicly open High Throughput Experimental Materials (HTEM) database currently contains more than 140 000 classified entries.^122^ Apart from easing the exploration of vast material spaces with their corresponding catalogue of structural, chemical and optoelectronic properties, the database is organized so as to potentially feed ML algorithms for the development of predictive models or identification of statistical trends. A similar open-data repository is available as well regarding solar fuels materials in the Materials Experiments and Analysis Database (MEAD).^123^ In OPV, public consortia such as the recently launched emerging-pv.org platform^10,124^ will be very useful in organizing and automatically updating a state-of-the-art database freely exploited by data scientists. In this case, top-performing photovoltaic devices of emerging technologies are carefully indexed, including as well the experimental details required to reproduce the corresponding devices and performance figures. These suites of classified individual experiments will complement very well the even richer computational screening compilations of materials such as that of the Harvard CEP.^40^

One of the first and most important usages of data mining is to search for potential correlations between features of the photoactive molecules employed in the active layer of the devices and their ultimate performance. As previously mentioned, such features are known as *descriptors*: they might include intrinsic optoelectronic properties such as frontier molecular orbitals energy levels and energy band gaps, as well as others related with chemical graphs such as molecular connectivity indices or number of heteroatoms. An extensive catalogue of computational tools are currently available to retrieve thousands of molecular descriptors from any molecule right from their simplified molecular-input line-entry system (SMILES) string.^118^ This particular discipline serves to establish *quantitative structure–property relationships* (QSPR) or *quantitative structure–activity relationships* (QSAR), and in the case of OPV we encounter several examples of successful correlation detection. Since the visual inspection of the databases to extract dependencies with molecular properties is far from being straightforward, ML models are employed to retrieve potential correlations as well. However, special attention must be paid to the curation of the indexed data when dealing with molecular modeling investigations,^125^ as the structural errors can have a detrimental effect on the predictive ability of the obtained models.^126^

Sahu, Ma and co-workers built a database consisting of *ca.* 300 small-molecule OPV devices retrieved from literature and applied ML models to draw potential QSPR correlations.^127,128^ In their data analysis, they included 13 different geometrical descriptors and optoelectronic magnitudes computed *via* DFT calculations as well as the experimentally measured device characteristics, such as *V*_oc_, *J*_sc_, FF and PCE, as target properties. For data interpretation, they tested up to five different ML techniques and found that random forest (RF) ensembles and gradient boosting regression trees (GBRT) performed significantly better when training predictive PCE models.^127^ Their results indicate that in small-molecule OPV devices containing either [6,6]-phenyl-C_61_-butyric acid methyl ester (PC_61_BM) or [6,6]-phenyl-C_71_-butyric acid methyl ester (PC_71_BM) as acceptors, the degeneracy of the frontier molecular orbitals,^127^ the LUMO and LUMO+1 levels of the donor, their alignment with those of the acceptor and the change in the dipole moment from the ground state to the first excited state of the donor molecule might have more importance than thought so far in determining the PCE.^128^

In the case of ternary blends, including as well fullerene derivatives as acceptor species, Min-Hsuan Lee constructed a photovoltaic performance dataset of 124 material combinations together with their corresponding frontier energy levels retrieved experimentally in the same publications.^129^ In this work, the predictive accuracy in terms of PCE of up to 5 different regression ML models and 6 classifiers was compared until identifying that RF model ensembles outperformed amongst the tested approaches. Interestingly, the LUMO level of the donor materials was found to be the most important parameter in determining the photovoltaic performance, in good agreement with previous data mining studies. That same year, a similar study was reported exploiting a database consisting of 135 donor:NFA pairs showing that, in this particular type of blends, the band gap of the NFA and the HOMO level of the donor are the most important parameters in drawing PCE predictions.^130^ In this case, a RF regressor was demonstrated to retrieve a coefficient of determination (*R*^2^) of 0.80 in the testing set.

More recently, Wu *et al.* collected the photovoltaic performance (*V*_oc_, *J*_sc_, FF and PCE) of 565 donor:acceptor pairs from the literature and tested the predictive accuracy for the PCE of a variety of ML algorithms.^131^ The study shows once again that RF models and GBRT outperform the investigation of the performance in polymer:NFA devices. However, in this study Wu *et al.* introduced an advanced segmentation of the input molecules into fragments, which were then combinatorially screened to propose new high-performing candidates and donor:acceptor pairs (Fig. 8b). They identified and synthesized novel materials demonstrating PCEs beyond 13%. Reassuringly, the same donor:acceptor pair was shown to reach a PCE as high as 16.5% in a different laboratory.^132^ These results illustrate that, on the one hand, data mining has an inherent drawback related with the consistency of the retrieved datasets, as these correspond to different material suppliers and manufacturers that follow a rich catalogue of protocols in distinct worldwide laboratories; and, on the other hand, that ML algorithms are powerful tools to realize molecular screening and propose potentially high-performing photovoltaic candidates. In particular, this property is called to enable the rapid identification of suitable photoactive candidates and donor:acceptor pair combinations motivating their ulterior synthesis and characterization.

Very recently, our group implemented a consistent pairing between high-throughput experimentation based on gradients and AI algorithms to extract predictive models for the photocurrent-composition dependence in organic solar cells.^26^ The approach starts by collecting large datasets consisting of hundreds of thousands of data points drawn from the systematic high-throughput screening of 15 distinct donor:acceptor pairs performed *via* 2D thickness-composition libraries. These are used to train either a Bayesian machine-scientist^133^ or distinct RF model ensembles. Surprisingly, we identify powerful predictive models for the photocurrent-composition space that employ two input parameters only, namely the electronic (or optical) band gaps of the materials under study. Similarly, Du, Lüer *et al.* fed a GPR algorithm with data consistently retrieved from their AMANDA automated research line and identified powerful predictive models in terms of efficiency and photostability for faster optimization of the blend under study.^99^ Given these results, the implementation of AI algorithms in conjunction with consistent experimental datasets is largely encouraged in both gradient-based and robotized high-throughput combinatorial studies.

**Fig. 9:**
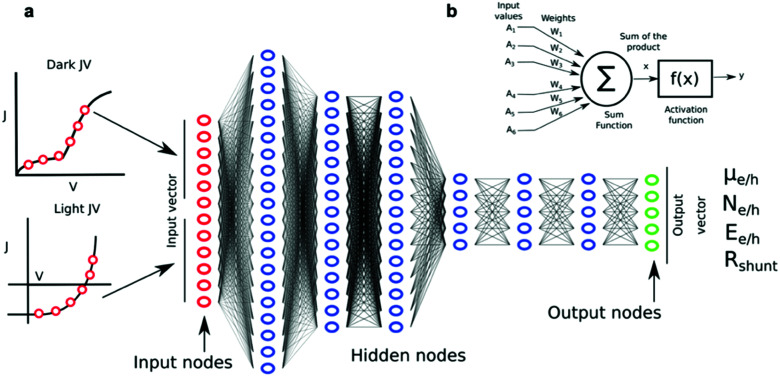
(a) A diagram of the neural network used by Majeed *et al.* to extract material parameters from the generated *JV* curves. Visible on the left-hand side of the image is the experimental (or simulated) data (*i.e.* dark and light *JV* curves), with the red dots on the curves representing the points at which the curves were sampled to form input vectors of length 12 for the neural network. This network has red input nodes, blue hidden layers, and green output nodes. Each output node corresponds to a device/material parameter such as charge carrier mobility (*μ*_e/h_) or trap density (*N*_e/h_). (b) Architecture of a single neuron. Figure adapted from ref. 134 with permission from John Wiley and Sons.

Finally, *deep learning* is gaining increased attention to draw predictive PCE models in combination with data mining,^119^ as well as to overcome the computational limitations ascribed to fitting complex analytical models in organic solar cells.^118^ Majeed *et al.* trained a dense neural network using synthetic data (20 000 sample points) generated by the Schockley–Read–Hall based drift-diffusion model.^134,135^ Their output layer of nodes included relevant microstructural parameters of the devices such as charge carrier mobilities, Urbach energies, trap densities, recombination time constants and contact and shunt resistances (Fig. 9). After successful training, the neural network was employed to extract such a catalogue of parameters using as inputs only the dark and light *JV* curves of the experimental devices. In this way, they investigate the origin of the improved performance in organic solar cells that are thermally annealed or processed with additives. In particular, their results show that the major improvements are related with an increased carrier mobility and reduced shunt resistance.

## Outlooks in high-throughput screening

OPV is lagging behind in terms of costs and stability, investigation on the use of green solvents and all-printed photovoltaic modules.^10^ With the PCE now approaching 20% in lab scale devices, performance is no longer a bottleneck in the OPV technology but the increase of operational lifetimes. For this reason, high-throughput stability studies^93,99,136^ are gaining attention and should be fostered in the next few years to complement material discovery. Environmentally-friendly ink engineering represents an additional aspect that could be exploited as well in high-throughput experimentation setups.^101^

**Fig. 10:**
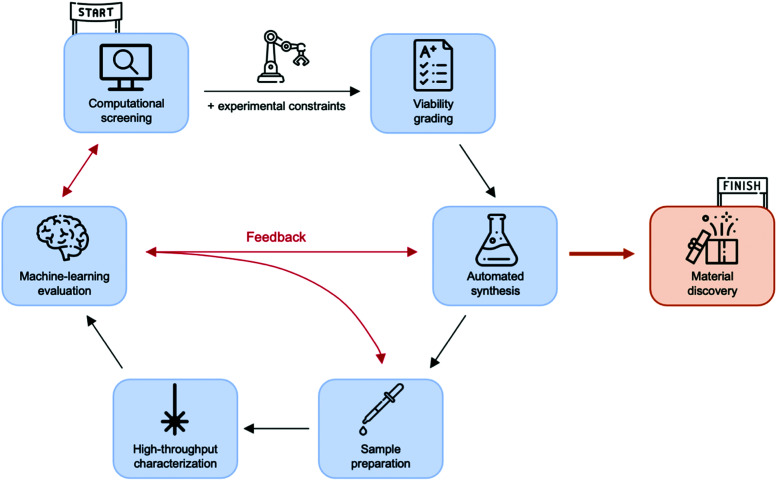
Workflow of a closed-loop approach towards autonomous material discovery. The loop starts by performing a first computational screening step of molecular candidates, which are evaluated in terms of computationally-accessible figures-of-merit such as PCE based on device models (*e.g.*, the Scharber model).^36^ The screening procedure is biased by the experimental limitations of the processing equipment (robotic arms) and eventually grades selected candidates based on their viability in terms of automated chemical synthesis. Then, automated sample preparation is combined with high-throughput characterization to rapidly generate experimental datasets and feed ML models. At this stage, AI is exploited to both analyse the experimental data and to orchestrate ulterior experimental planning *via* feedback loops with the synthetic, processing and even computational screening steps. The loop should ideally end when a molecular candidate can be automatically synthetised while matching the desired target properties.

On the other hand, autonomous experimentation in self-driven laboratories are envisioned by many as the next-generation, all-in-one high-throughput screening platforms in the upcoming decade.^103,104,137,138^ These fully integrated (closed-loop) workflows (Fig. 10) are expected to start with a computational virtual screening step of molecular candidates based on certain figures-of-merit, which should be accessible computationally. The procedure follows then by a careful grading based on the viability of their chemical synthesis, including also a bias determined by the limitations of the employed equipment (robotic arms in this case).^137^ After material synthesis and sample preparation, self-driven laboratories require high-throughput characterization approaches such as those based on hyperspectral imaging,^27^ combining as well ML methods, to rapidly construct an approximation of the landscape under study and propose new experiments. Finally, the upcoming experiments are orchestrated with the help of AI in a closed feedback loop until matching the target property, yield or performance.

However, it is also considered that the high-throughput experimental synthesis and characterization are the main bottlenecks of the process.^137^ Accordingly, these are subjected to intense research; as an example, novel data-driven experiment planning algorithms such as the Bayesian-based Gryffin approach^139^ have been exploited very recently to drive the synthesis of NFAs at a substantially high discovery pace.

## Conclusions

Multivariate optimization problems in OPV and related thin-film technologies have traditionally been handled using time- and resources-expensive methodologies such as those based on the manual prototyping of tens or hundreds of samples. The adoption of higher throughput screening approaches is encouraged to accelerate the discovery of novel materials and understanding of their structure–property relationships. In this review, we first summarized some of the main efforts towards computational screening of materials, which result in the *in silico* evaluation of the potential of thousands of compounds. Then, we presented two high-throughput experimental branches to face this kind of problems in research laboratories: gradients and robots.

Gradient-based approaches rely on the fabrication of continuous parametric libraries, either from evaporation sources or directly from solution, to explore the multivariate space in an *analog* fashion. This approach accordingly requires fast acquisition, large-area characterization techniques to leverage the combinatorial nature of continuous libraries. Conversely, robotized laboratories delegate the manufacturing and characterization entirely to robot arms, which guarantee an unbeatable yield and batch-to-batch reproducibility during the *digital* exploration of the multivariate space in discrete steps. These two methodologies are compared in terms of throughput and raw material requirements, including the emerging self-driven laboratories where AI algorithms control the actuation and experimental planning in robotized setups. Finally, we evaluate the unique capabilities that high-throughput screening experimental approaches have in generating large datasets (*big data*). These render useful to extract statistically meaningful trends from the experiments, draw consistent rationale and promote further material discovery through synergy with generative and predictive ML models.

## Author contributions

All authors wrote and discussed about the manuscript.

## Conflicts of interest

There are no conflicts to declare.

## Supplementary Material

EE-014-D1EE00559F-s001
