# Supplementary material for: Accelerating organic solar cell material's discovery: high-throughput screening and big data
Source: Energy Environ Sci. 2021 Apr 23;14(6):3301–22. doi: 10.1039/d1ee00559f (PMC8209551; doi:10.1039/d1ee00559f)
Supplement: EE-014-D1EE00559F-s001 [file EE-014-D1EE00559F-s001.pdf]

Electronic Supplementary Material (ESI) for Energy & Environmental Science.
This journal is © The Royal Society of Chemistry 2021

Supplementary Information for

Accelerating organic solar cell material's discovery: high-throughput screening and *big data*

Xabier Rodríguez-Martínez[a], Enrique Pascual-San-José[a] and Mariano Campoy-Quiles*[a]

[a] Institut de Ciència de Materials de Barcelona, ICMAB-CSIC, Campus UAB, 08193, Bellaterra, Spain.

* Author to whom correspondence should be addressed: m.campoy@csic.es

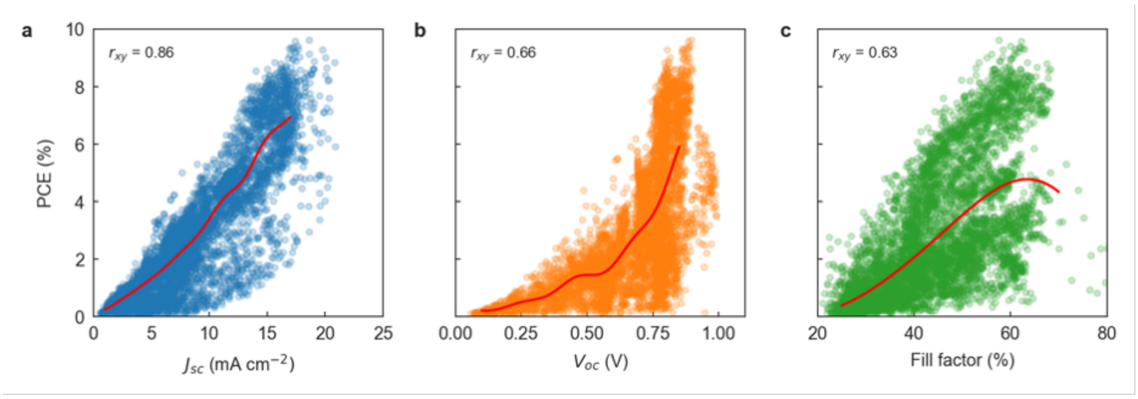

Figure S1. PCE correlations drawn from our in-house database of >5,000 OPV devices for (a) J_{sc}, (b) V_{oc} and (c) FF. The corresponding Pearson correlation coefficients (r_{xy}) are also included. Solid red lines represent support vector machine (SVM) models applied to the scattered data, which serve to draw the main statistical trendlines.

Table S1. Comparative figures in terms of cost for different experimentation approaches, namely one-variable-at-a-time (discrete sampling) and 1D/2D high-throughput experimentation based on lateral gradients. The reader should note that in this case example only the costs in terms of OPV materials have been considered; the energy requirements in the overall process (and their associated costs) have been omitted for the sake of simplicity. The values in brackets correspond to the percentage with respect to the overall price.

	**Discrete sampling**	**1D discrete gradients**	**2D continuous gradients**
Problem dimensionality	1D	2D	2D
No. data points per sample	6	24	24.000
No. non-equivalent data points per sample	1	12	24.000
ITO substrate	2.30€ [a] (24.7%)	5.83€ [a] (45%)	2.88€ [b] (29.1%)
Electron transport layer materials[c]	0.20€	0.20€	0.20€
Donor (PBDB-T)[d]	2.28€	2.28€	2.28€
Acceptor (ITIC-4F)[e]	3.09€	3.09€	3.09€
Solvent (CB)[f]	0.03€	0.03€	0.03€
Subtotal Active layer	5.40€ (58%)	5.40€ (42.3%)	5.40€ (54.6%)
Hole transport layer materials (PEDOT[g]/MoO_3[h])	0.10€ [g]	0.09€ [h]	0.09€ [h]
Top electrode materials (Ag)[i]	0.13€	0.13€	0.13€
Encapsulation[j]	1.20€ (12.9%)	1.20€ (9.4%)	1.20€ (12.2%)
Sample cost	9.32€	12.77€	9.90€
Cost per non-equivalent data point	9.32€	1.06€	0.0004125€
Cost of 6-samples batch	55.92€	76.04€	59.4€
Non-equivalent data points in a 6-samples batch	6	72	144000

[a] Cost estimated from Ossila webpage. Pixelated ITO substrates (6 devices) (https://www.ossila.com/products/oled-ito-substrates?variant=1200244789) (12 devices) (https://www.ossila.com/products/pv-and-oled-scale-up-substrates?variant=6462656641).
[b] Cost estimated from Ossila webpage. Unpatterned ITO glass substrates (https://www.ossila.com/products/ito-glass-substrates-unpatterned?variant=31274504978528).
[c] Cost estimated from Avantama webpage. Avantama N-10 (440 CHF = 407€/100 mL) and 50 μL per sample.
[d] Cost of PBDB-T from Sigma Aldrich (https://www.sigmaaldrich.com/catalog/product/aldrich/901099?lang=es&region=ES), 480€/100 mg, each sample has 0.6 mg.
[e] Cost of ITIC-4F from Sigma Aldrich(https://www.sigmaaldrich.com/catalog/product/aldrich/901423?lang=es&region=ES), 515€/100 mg, each sample has 0.6 mg.
[f] Cost of chlorobenzene (CB) from Sigma Aldrich (https://www.sigmaaldrich.com/catalog/product/sial/284513?lang=es&region=ES), 104€/250 mL, each sample has 80 μL.
[g] Cost of PEDOT Al 4083 from Ossila (https://www.ossila.com/products/pedot-pss?variant=30366225236064), 203€/100 mL, each sample has 20 μL.
[h] Cost of MoO_3 pellets from Lesker (https://www.lesker.com/newweb/deposition_materials/depositionmaterials_evaporationmaterials_1.cfm?pgid=moo3), 518€/100 g, each sample is assumed to have 100 mg divided by the number of evaporated devices at the same time (6).
[i] Cost of Ag pellets from Lesker (https://www.lesker.com/newweb/deposition_materials/depositionmaterials_evaporationmaterials_1.cfm?pgid=ag1), 190€/100 g, each sample is assumed to have 400 mg of Ag divided by the number of evaporated devices at the same time (6).
[j] Cost of encapsulation resin (DELO), 183€/70 g, 0.2 g of resin per sample is assumed. Cover slides (Ossila) 68€/100 units (https://www.ossila.com/products/encapsulation-coverslips?variant=31488550568032).
